# Supplementary material for: Network impact of a single-time-point microbial sample
Source: PLoS One. 2024 May 30;19(5):e0301683. doi: 10.1371/journal.pone.0301683 (PMC11139317; doi:10.1371/journal.pone.0301683)
Supplement: Attachment — Submitted filename: Response to Reviewers.pdf [file pone.0301683.s001.pdf]

PONE-D-23-16744, Network impact of a single-time-point microbial sample, PLOS ONE

Response to Reviewers

Reviewer #1: The manuscript "Network impact of a single-time-point microbial sample" reports new methods of analysis of microbial community composition. Prior work has analyzed how microbial community composition changes over time. Correlations within such networks reveal the interactions between different microbial species and the impact of these interactions on changes in species abundance over time. However, in many relevant contexts, it is difficult to take time-course measurements of community composition. The reported methods attempt to solve this problem by comparing the community composition at one time point with the characteristic composition of similar communities from prior measurements. The hope is that such comparisons might identify when a community is abnormal, in that the pattern of species abundances are not expected given the underlying interaction network in the comparison samples. Here data used for these comparisons are generated using generalized Lotka-Volterra models. Data generated from the same matrix of growth rate and interaction parameters are combined to create the reference cohort. This standard dataset is then rearranged to approximate individual samples that should disagree with the model used to generate the standard cohort. Several different analysis schemes are compared, with distance-based analysis representing a standard approach.

Overall the findings of the paper are interesting. The authors clearly demonstrate that the proposed analytical methods do reveal samples that differ from the reference cohort. Minor changes should be made to improve clarity of the manuscript, as described below.

1. The notation is the paper is at times confusing, particularly the use of i and j to mean multiple things. For example, in equation 1 i and j are different species in the same community, whereas later around line 99 j is a species and i is a sample number. Later on line 131 k indicates the sample number. Particularly in the paragraph starting on line 129 it is not clear for calculations of N_ij and p_ij if species abundances are compared only within a community or between different community samples. More explanation and consistent notation would help.

We thank the reviewer for this very important comment. We revised all the mathematical notations in the manuscript to keep coherent definitions of the indices. Specifically, along the revised manuscript, i and j represent species within the same community and k represent a sample within a cohort.

2. For the semi-supervised case C = 0.1, which means 90% of species combinations are not interacting. However for the supervised case C = 0.8, which means only 20% of species combinations are not interacting. It is unclear if this seemingly large change greatly impacts the ability of the metrics proposed to identify abnormal population ratios, but a note probably should be added explaining the need for the increase in C when switching to the supervised case.

The reason for increasing the number of interactions (larger C) and decreasing their strength (lower \sigma) in the case of supervised classification is to minimize the effect of the inter-species interactions on the characteristic abundance of the individual species. This is relevant in the case of supervised classification since the two cohorts are generated using two GLV models that differ in their interaction matrices. This may lead to a case where the same individual species has different characteristic abundance profiles even when they have the same growth rate, if, for example, one of them has many positive interactions (affected positively by other species) while the other has many negative ones. By choosing large C and small sigma this effect is statistically reduced.

We explain this in the revised manuscript (lines 268-272).

> *Here, we choose a large value of C and weak interaction strength (low value of σ) to reduce the accumulated effect of the inter-species interactions on the characteristic abundance of the individual species. In other words, when all species have many weak interactions, we expect that, on average, the contributions of the positive and negative interactions on the species abundance will cancel out.*

3. On line 246 it is stated that “a large reference cohort may mask the relative network impact of a single sample”, but that seems like a failure of the analytical approach not a general problem of having too much initial data. Having more cohort data should improve the ability to identify abnormal sample, not make it more difficult. Is there a version of this analyze the corrects for the size of the reference cohort?

We completely agree with the reviewer that having a large number of reference samples is overall advantageous. We added an explanation that the optimal range of m found in Fig. 4 can help to divide the large reference cohort into several smaller sub-groups and calculate ΔS or ΔW over them.

This is explained in the revised version of the manuscript (lines 248-250).

> *When considering both ΔS and ΔW, it is evident that in our simulations the distributions for the cases of $m=50$ and $m=100$ have the highest AUC values. This suggests that there is an optimal range of m values for maximizing the separability between real and shuffled profiles using ΔS and ΔW, where the analysis of a small reference cohort can result in a noisy network, and a large reference cohort may mask the relative network impact of a single sample. The case of too large reference cohort for direct application of ΔS or ΔW can be effectively mitigated by calculating them over sub-groups of the reference samples, whose size should be chosen according to the optimal range of m.*

4. It seems like there would be some value to testing the ability of these metrics to identify samples generated from a GLV model using a different growth rate and interaction matrix, as

opposed to only shuffling data generated from the same model parameters. Likely in real contexts, changes in external conditions or species genetic variation may modify these matrices, which leads to changes in the patterns of species abundance for some samples/patients.

The case of two cohorts that differ in the species growth rates, while representing many realistic scenarios, does not require a network impact approach but rather a simple abundances analysis of the individual species. Thus, to focus on the effect of the inter-species interaction we intentionally generate cohorts that share the same growth rates.

This is explained in the revised version of the manuscript (lines 263-266).

> *All GLV models are created using the same set of r_i values, such that the characteristic abundance of each species is preserved across the different models. The alternative case, where the individual species have different growth rates, and consequently, differential abundance profiles, can be easily classified using distance-based analysis of the abundance profiles, without the need to assess the species-species interrelations.*

Minor comments:
Page 1: “vitamins produce” should probably be vitamin production.

Fixed.

On pages 3 and 5 the phrase “sum to a unit” is used. Sum to one is more familiar to me, although sum to a unit may be acceptable.

Fixed.

On line 101 it states $X_{ij} := X_{ij}$ / sum X_{ij}. Isn’t that a recursive definition of X_{ij}?

We explicitly explain this in the revised manuscript (lines 100-102).

> *…Finally, we normalize the samples such that the abundances of all species in each sample sum to one, i.e., in terms of computational notations, $X_{ki}:=X_{ki}/\sum_i X_{ki}$, where $:=$ is the assignment expression operator.*

On line 112, I believe it should be: k is a random integer $1 <= k <= m$ not $1 <= k <= i$.

This range of k values (changed to v when comment 1 was addressed) are between 1 and m since the algorithm needs to choose each product of the vector once.

Line 223: “This result arises a practical question” should be “This result suggests a practical question” or “This result brings to light a practical question”.

Fixed.

We again thank the reviewer for thoroughly reviewing our manuscript and for their instructive and helpful comments.

Reviewer #2: Reviewed the manuscript titled "Network impact of a single-time-point microbial sample". The authors present a method to estimate the divergence between a single sample and a cohort of samples that may represent different conditions, then compare this procedure with more traditional distance-based measures, and finally show two different approaches to use their method. The methods are technically sound and the data support the conclusions. The statistical analysis is appropriate. Both the data and code used in this manuscript are readily available in the indicated repository. The language used in the manuscript is clear and the text looks well prepared.

Recommended minor revisions to improve the manuscript for readability and attend a suggestion on the presentation of the story: The authors give much weight to the human microbiome and the application of their method for personalized medicine. Maybe presenting the problem being addressed by this method in a wider context could attract researchers from other fields like biotechnology (population dynamics within bioreactors), agriculture (state of rhizospheric communities), and microbial ecology in general (impact of climate change in a specific system). Besides, the applications on human microbiome research are barely mentioned in the conclusions. It is understandable if the authors want to keep the focus in the human microbiome, if that is the case they should further discuss the implications for this field.

We thank the reviewer for this constructive comment. We added a paragraph in the revised Conclusion section mentioning that the network impact approach can be directly applied to other microbial ecosystems, beyond the human microbiome. (lines 359-365).

More specific suggestions are given below:
- Remove the expression of the GLV model from the introduction and refer to methods or Figure 1

We agree with this suggestion and modified the Introduction and the Methodology sections accordingly.

- Maybe add a visual explanation for the three parameters in Figure 1 or 2

The visual explanation of ΔS and ΔW are shown in Fig1. An indication was added to the figure.

- Line 211, replace "if"

Fixed.

- Text in all the plots is too small, please enlarge it

Fixed.

- Text in Figure 3 looks narrow, please enlarge/change the typography

Fixed.

- Rename the current "Conclusion" section to "Discussion" and add a concise "Conclusion" section with the main take-home messages

Done.

We again thank the reviewer for thoroughly reviewing our manuscript and for their instructive and helpful comments.
